# The crystal structure of *Escherichia coli *TdcF, a member of the highly conserved YjgF/YER057c/UK114 family

**DOI:** 10.1186/1472-6807-7-30

**Published:** 2007-05-16

**Authors:** Julia D Burman, Clare EM Stevenson, R Gary Sawers, David M Lawson

**Affiliations:** 1 Department of Biological Chemistry, John Innes Centre, Norwich NR4 7UH, UK; 2 Department of Molecular Microbiology, John Innes Centre, Norwich NR4 7UH, UK; 3 Department of Biology and Biochemistry, University of Bath, Bath BA2 7AY, UK; 4 Max-Planck-Institut für Terrestrische Mikrobiologie, Karl-von-Frisch Strasse, D-35043 Marburg, Germany

## Abstract

**Background:**

The YjgF/YER057c/UK114 family of proteins is widespread in nature, but has as yet no clearly defined biological role. Members of the family exist as homotrimers and are characterised by intersubunit clefts that are delineated by well-conserved residues; these sites are likely to be of functional significance, yet catalytic activity has never been detected for any member of this family. The gene encoding the TdcF protein of *E. coli*, a YjgF/YER057c/UK114 family member, resides in an operon that strongly suggests a role in the metabolism of 2-ketobutyrate for this protein.

**Results:**

We have determined the crystal structure of *E. coli *TdcF by molecular replacement to a maximum resolution of 1.6 Å. Structures are also presented of TdcF complexed with a variety of ligands.

**Conclusion:**

The TdcF structure closely resembles those of all YjgF/YER057c/UK114 family members determined thus far. It has the trimeric quaternary structure and intersubunit cavities characteristic of this family of proteins. We show that TdcF is capable of binding several low molecular weight metabolites bearing a carboxylate group, although the interaction with 2-ketobutyrate appears to be the most well defined. These observations may be indicative of a role for TdcF in sensing this potentially toxic metabolite.

## Background

The YjgF/YER057c/UK114 family of proteins is highly conserved and is found in bacteria, archaea and eukarya, and has recently been discovered in plants [1]. Despite their ubiquity, no function for any member of the class has been clearly defined, although evidence has been obtained which suggests that, at least in certain microbes, there is a link with isoleucine metabolism [2-5]. All members of the class studied in detail are homotrimeric with subunits of 120–130 amino acids in length. The structures of several YjgF family members have been determined, the first two of which were published in 1999, namely YjgF from *Escherichia coli *[6] and YabJ from *Bacillus subtilis *[7]. These structures revealed a cavity, located at the subunit interfaces and decorated by seven totally conserved amino acids within the family [8]. This finding immediately suggested that this cavity could represent a binding site for a substrate or ligand. Indeed, the site of substitution for the mercury derivative used to solve the structure of YabJ was Cys-104, a reasonably well-conserved residue that lines this pocket. In total, the structures of 14 homologues have now been deposited in the Protein Data Bank (PDB) and these are summarised in Table 1, only one of which shows a biologically significant ligand bound in this site. This is the structure of the human protein hp14.5 [9], which contains at least one benzoic acid molecule per site forming bi-dentate interactions between its carboxylate moiety and the guanidinium group of the strictly conserved Arg-107. This structure contains three trimers per asymmetric unit and four of the nine possible sites also show an additional, weakly bound, benzoic acid molecule adjacent to the first, oriented in one of two possible conformations. It is notable that these crystals were obtained in the presence of 0.3 M sodium benzoate, and thus the biological relevance of this interaction may be questionable.

Recently, nuclear magnetic resonance spectroscopy of the HI0719 protein from *H. influenzae *[8] revealed that 2-ketobutyrate and analogues of its cognate enamine, interacted with this cavity, suggesting that at least some of these proteins might bind keto acids. This finding supports a number of *in vivo *observations, which have pointed towards a role for some family members in L-isoleucine metabolism [2-5].

During isoleucine biosynthesis L-threonine is deaminated to 2-ketobutyrate by the IlvA protein. In yeast a mutation in one of the YjgF/YER057c/UK114 family paralogues results in isoleucine auxotrophy and impaired mitochondrial maintenance [4,10,11]. In *Salmonella enterica *strong evidence has been provided [3] that shows when the *yjgF *gene is mutated, the specific activity of the final enzyme (IlvE) on the biosynthetic pathway to L-isoleucine is significantly reduced, suggesting that YjgF acts at a post-translational level in controlling IlvE activity. More recent data [12] reaffirm the conclusion that YjgF interacts with a specific metabolite. Taken together, these data suggest that at least some members of this large family of proteins might act as sensors of cellular 2-ketobutyrate levels. Accumulation of 2-ketobutyrate results in toxicity towards cells and this has been proposed to result from competition with 2-ketoisovalerate, which is a precursor in coenzyme A biosynthesis [13]. Consequently, cells may well have evolved a mechanism for sensing 2-ketobutyrate levels and altering metabolism to degrade this potentially toxic intermediate.

**Figure 1:**
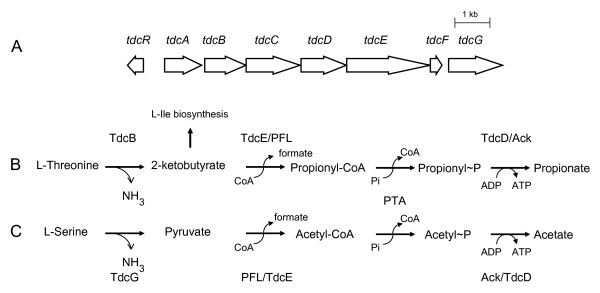
**The *tdc *operon and the pathways for degradation of L-threonine and L-serine**. Schematic representation of the organisation of the *tdc *operon on the chromosome of *E. coli *and the function of the respective gene products. (A) shows the *tdc *operon [15], where the *tdcA *and *tdcR *gene products are transcriptional regulators, while the *tdcC *gene product is L-threonine/L-serine permease [30]. Also shown are the metabolic pathways for the anaerobic degradation of (B) L-threonine and (C) L-serine, where: Ack = acetate kinase; PFL = pyruvate formate-lyase; PTA = phosphotransacetylase.

Degradation of 2-ketobutyrate to propionate occurs via propionyl-CoA and propionyl-phosphate intermediates with the generation of ATP [14,15] (Figure 1). This pathway is analogous to the fermentative metabolism of acetyl-CoA. In enteric bacteria, coenzyme A-dependent cleavage of 2-ketobutyrate can occur both aerobically via pyruvate dehydrogenase and anaerobically via the glycyl radical enzyme pyruvate formate-lyase. *Escherichia coli *also has a dedicated pathway for the anaerobic degradation of L-threonine or L-serine that is encoded by the *tdc *operon [15,16] (Figure 1). The sixth gene of the *tdc *operon encodes a protein, TdcF, that is a member of the YjgF/YER057c/UK114 family. The location of this gene within the *tdc *operon is strongly suggestive of a role in the degradation of 2-ketobutyrate. However, all enzymic steps on this pathway can be accounted for by other enzymes encoded within the *tdc *operon, together with phosphotransacetylase [15], suggesting that TdcF might have a different function related to 2-ketobutyrate metabolism. In this study, we present crystal structures of TdcF in complex with a variety of ligands, most notably 2-ketobutyrate.

## Results

### Structure determination of TdcF

**Figure 2:**
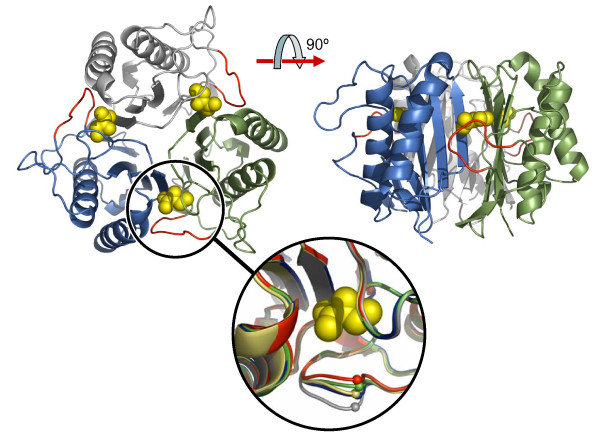
**The overall structure of *E. coli *TdcF**. Ribbon representation of the *E. coli *TdcF structure. Shown is the 2-ketobutyrate-bound form with the individual subunits coloured green, blue and grey; the 2-ketobutyrate ligands are shown in space-filling representation in yellow. Part of the loop between β1 and β2 that moves depending on the status of the ligand-binding pocket is shown in red for each subunit. The inset shows the extent of this movement in the various states, where: grey = empty site; blue = ethylene glycol-bound; yellow = serine-bound; green = propionate-bound; red = 2-ketobutyrate-bound. The small spheres indicate the variation in the position of the Cα atom of Ile-14, being the residue that is displaced the most (maximum displacement 4.2Å). It is notable that the loop is poorly ordered in the empty site and best resolved in the in the 2-ketobutyrate-bound site, where Ile-14 makes non-bonding interactions with the ligand. Figure generated using *PyMOL *[31].

The first crystal structure of TdcF was determined at 2.35 Å resolution by molecular replacement using the structure of the paralogue YjgF [6] as a template, as described previously [17]. There is one homotrimer per asymmetric unit, with the subunits tightly packed together around a non-crystallographic three-fold axis, as a result burying around 2000 Å^2 ^of accessible surface per monomer, as estimated by the Protein Interfaces, Surfaces and Assemblies (PISA) server [18]. The trimer resembles an oblate spheroid with a pole-to-pole distance of approximately 45 Å and an equatorial diameter of approximately 55 Å (Figure 2). As expected, the TdcF structure is closely similar to those of the other YjgF/YER057c/UK114 family members that have been determined previously, with root-mean-square-displacements (rmsd) in Cα positions not exceeding 2.0 Å after superposition (see Table 1). As the fold has been described extensively elsewhere [6-10,19,20] only brief details will be given here. Each 14 kDa subunit comprises a single domain that contains a predominantly anti-parallel, six-stranded β-sheet against which are packed two short α-helices. The connecting loops on the outer surface of the trimer are long in comparison to the loops positioned at the centre of the protein, which are much shorter and tighter. The core of the trimer contains a triangular barrel-like structure, which is formed from twelve β-strands, with four donated from each monomer (Figure 2). This central cavity is filled with some 20 ordered water molecules. The six α-helices, two per subunit, decorate the periphery of the molecule.

The most significant structural features are the three symmetry-related solvent-accessible clefts, located at the interfaces between pair of subunits, close to the "equator" of the trimer. The clustering of well-conserved amino acids in and around these sites strongly suggests that they are functionally important. The three sites, which we shall refer to as sites A, B and C, are not crystallographically equivalent, and are thus subject to different crystal packing environments, with site A being the most occluded by neighbouring trimers, and site C being the most solvent exposed.

### The binding of ethylene glycol

**Figure 3:**
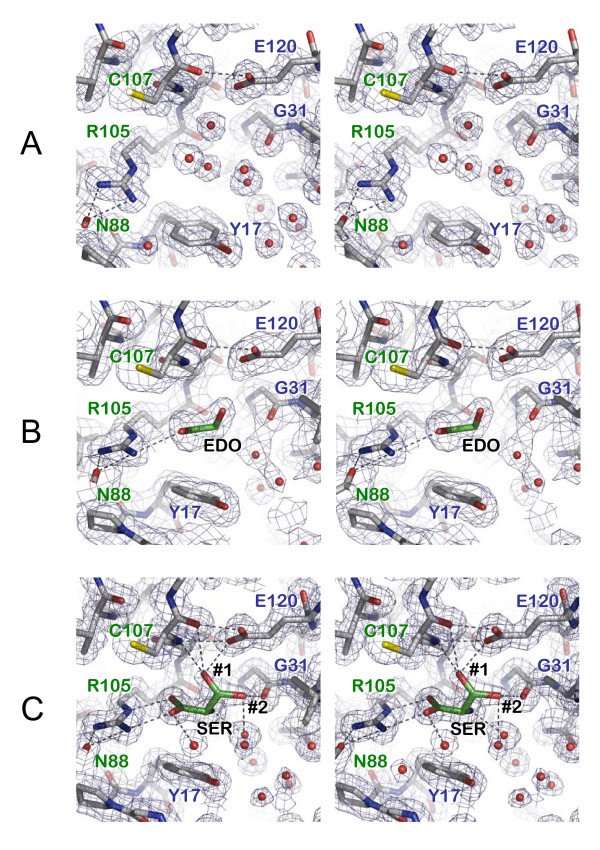
**The ligand-binding pocket of TdcF in the empty, ethylene glycol-bound and serine-bound states**. Stereoviews showing *2mF*_*obs *_- *dF*_*calc *_electron density maps contoured at approximately 1 sigma superposed on TdcF binding pockets in the following states: (A) empty (1.6 Å resolution); (B) with ethylene glycol bound (2.35 Å resolution); (C) with serine bound (1.6 Å resolution), where #1 and #2 denote alternate conformers for the Oγ. Important hydrogen bonds are shown as dashed lines. Residues from different subunits are labelled green and blue, respectively. In all cases, the side-chain of Arg-105 makes a bi-dentate interaction with the Oδ1 of Asn-88, which is just visible in the left foreground; and Oε1 of Glu-120 makes an inter-subunit hydrogen bond with the carbonyl oxygen of Cys-107. Throughout this figure, the view is similar to that seen in the inset of Figure 2. Figure generated using *PyMOL *[31].

The original 2.35 Å resolution as-isolated X-ray data set was collected at 100 K using a cryoprotectant solution containing 20% (v/v) ethylene glycol. During model building and refinement, it became apparent that whilst the electron density in site A was consistent with ordered water molecules, a more substantial region of elongated density was present in both sites B and C. This could be modelled convincingly as a single ethylene glycol molecule in each of the two sites making a single hydrogen bonding interaction with the side chain of Arg-105 (Figure 3B). It was noted that the central part of the loop connecting β1 and β2, which delineates one side of the cleft and bears the conserved residue Tyr-17, had elevated temperature factors, with Ile-14 being poorly defined in the electron density maps. This was especially true for the loop adjacent to site A, which adopted a slightly more open conformation with respect to the loops in sites B and C that were partially closed over the ligand (see Figure 2). Only one other structure of a YjgF/YER057c/UK114 homologue, that of TM0215 (PDB accession code 2B33) from *Thermotoga maritima*, reports the use of ethylene glycol; although three molecules were resolved in this structure, they were all bound at surface sites away from the conserved ligand-binding pocket. Since ethylene glycol makes only a single hydrogen bond with TdcF, it is likely that it binds with low affinity, and it is only seen in the structure because of its high concentration in the cryoprotectant.

### The binding of a hydroxy amino acid

In all subsequent X-ray data collections, to preclude competition between ethylene glycol and any potential ligands for TdcF, ethylene glycol was substituted with PEG 400 in the cryoprotectant solution. A data set was collected to 1.6 Å resolution without the addition of any ligand, in order to visualise the structure of ligand-free TdcF. To our surprise, however, site C was clearly occupied by something other than water molecules. Given the resolution and quality of the electron density, it was possible to place atoms into the density with some confidence. Indeed, a good fit was achieved with a threonine residue, with the carboxylate making a bi-dentate salt-bridge to the side-chain of Arg-105, the amino group hydrogen-bonding to the carbonyl oxygen of Arg-105, and the hydroxyl group hydrogen-bonding to both the amino group and the carbonyl oxygen of Cys-107, as well as to the side-chain of Glu-120. However, the density for the side-chain of the threonine was noticeably weaker than for the rest of the residue and the temperature factors were marginally higher for these atoms after refinement. An alternative interpretation, with serine as the ligand with its side-chain in two alternative conformations of equal occupancy, gave a slightly better fit (Figure 3C). Moreover, the second side-chain conformation provides an additional hydrogen bond to the carbonyl oxygen of Gly-31. Although a fully ligand-free TdcF structure was not obtained, this model did provide details of a vacant pocket (sites A and B) at high resolution (Figure 3A).

### The binding of 2-ketobutyrate

**Figure 4:**
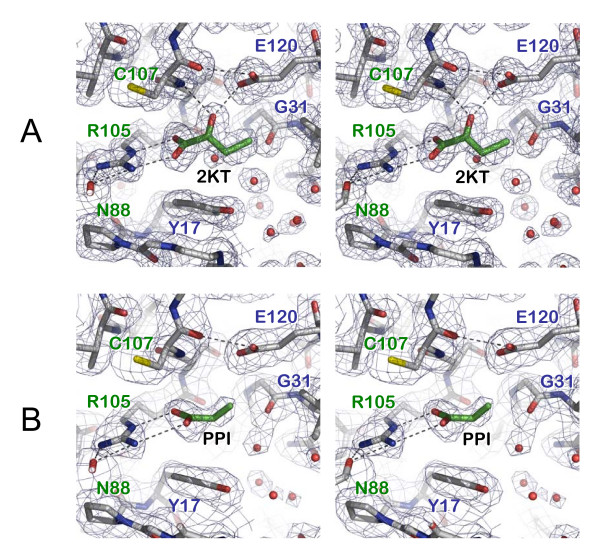
**The ligand-binding pocket of TdcF in the ketobutyrate-bound and propionate-bound states**. Stereoviews, prepared as for Figure 3, showing TdcF binding pockets in the following states: (A) with 2-ketobutyrate bound (1.6 Å resolution); (B) with propionate bound (2.45 Å resolution).

A further data set was collected to 1.6 Å resolution, this time from a crystal soaked in 1 mM ketobutyrate. The resultant electron density maps showed clear density for a 2-ketobutyrate ligand in each of the three binding sites (Figure 4A). As with the serine ligand, the carboxylate moiety of 2-ketobutyrate makes a bi-dentate salt-bridge to the side-chain of Arg-105, whilst the keto group forms two hydrogen bonds, one with the amino group of Cys-107, and the other with the carboxyl group of Glu-120. However, in order to provide a hydrogen for the latter interaction, the ligand would need to bind in the less stable enol form (see Figure 7). The significance of this observation is not clear at present. Curiously, there is a water molecule adjacent to the 2-ketobutyrate on the *re*-face of the enol moiety and approximately 2.8 Å from C_2_.

### The binding of propionate and other ligands

After soaking a crystal in 1 mM propionate, X-ray data to 2.45 Å resolution were collected. Sites B and C were clearly occupied with a ligand, and a propionate molecule could be placed with confidence in both. Again, the carboxylate group was salt-bridged to Arg-105 (Figure 4B).

Soaking experiments were also performed using L-threonine and L-serine, and data sets to 2.8 Å resolution were subsequently collected. In both cases, an area of positive difference electron density was present in site C that could be consistent with an amino acid, but further interpretation was not possible due to the poor resolution and quality of the data (not shown). A crystal soaked in propionyl-CoA did not survive the process, raising the possibility that the specific binding of this ligand induces a conformational change that disrupts the crystal lattice. Unfortunately, limited crystal availability precluded further soaking experiments.

### Roles of the conserved residues

Members of the YjgF/YER057c/UK114 family are characterised by seven totally conserved amino acids [8], all of which line the inter-subunit ligand-binding pocket. These are Tyr-17, Gly-31, Asn-56, Asn-88, Arg-105, Pro-114 and Glu-120 (Figure 7). The importance of Arg-105 is clear as it hydrogen-bonds to all ligands observed in the TdcF crystal structures, as well as to the benzoic acid moiety seen in the hp14.5 structure [9]. Glu-120 also forms key hydrogen bonds to the 2-ketobutyrate and to the putative serine ligand. In addition, Glu-120 hydrogen-bonds to the main-chain carbonyl of residue 107 across the subunit interface and therefore may be important for maintaining the structural integrity of the pocket. Asn-88 does not interact directly with any ligand, but may help to correctly orient Arg-105 through two hydrogen bonds, and in so doing also help to maintain the structure of the pocket. Tyr-17 and Pro-114 form non-bonding interactions with the ligands, whilst Gly-31 appears to be conserved purely on steric grounds: any residue with a side-chain would clash with other key residues, Glu-120 in particular. The function of Asn-56 is less clear as it lies some 9 Å away from the 2-ketobutyrate. It could play a role in mediating access to the pocket or may be involved in the binding of some other as yet unidentified ligand.

### Covalent modifications of TdcF

In the two structures determined at 1.6 Å resolution, inspection of the Fo-Fc electron density maps showed that, in all copies of Cys-36, the Sγ was surrounded by three peaks of positive density, that were too close to the atom to represent water molecules. This was modelled as a fully oxidised cysteine ie. cysteine sulfonic acid (Cys-SO_3_H), which gave a good fit to the electron density after refinement (Figure 6A). It was subsequently shown that cysteine sulfonic acid refined well at these positions in the two lower resolution structures as well. This modification is currently present in some 38 entries in the Protein Data Bank but is likely to be present, though undetected, in other structures determined at medium to low resolution. The fact that the residue is fully oxidised suggests that it is unusually reactive, but since this modification is generally irreversible, it is most likely of no biological significance. Furthermore, this residue is highly variable in the YjgF/YER057c/UK114 family, and it occurs in a surface loop of the protein, the Sγ being approximately 15 Å away from the nearest 2-ketobutyrate.

A further modification was apparent at Lys-58 in just the A chain of the 2-ketobutyrate-bound structure. This was modelled as a carboxylated lysine in two alternative conformations (Figure 6B). This modification occurs in over one hundred PDB entries, and in some cases has a well-defined functional role, e.g. in coordinating a metal ion in the active centre of dihydropyrimidinase (eg. PDB accession code 2FTW) [21]. Coincidentally, one occurrence of this modification is seen in the active site of transcarboxylase 5S subunit adjacent to a bound 2-ketobutyrate molecule (PDB accession code 1RR2) [22]. Although better conserved than Cys-36, this residue is located on the surface in helix 1 with its Cα approximately 15 Å away from the nearest 2-ketobutyrate, and thus is unlikely to be functionally relevant. Moreover, no evidence was seen for this modification in any of the other TdcF structures, although it is possible that this was not seen elsewhere due to disorder. Neither the Cys-36 nor the Lys-58 modifications could be detected by mass spectroscopy on freshly prepared sample or dissolved crystals (data not shown), which suggests that they may have arisen as artefacts after harvesting the crystals, and further supports the conclusion that they are not biologically important.

## Discussion

**Figure 5:**
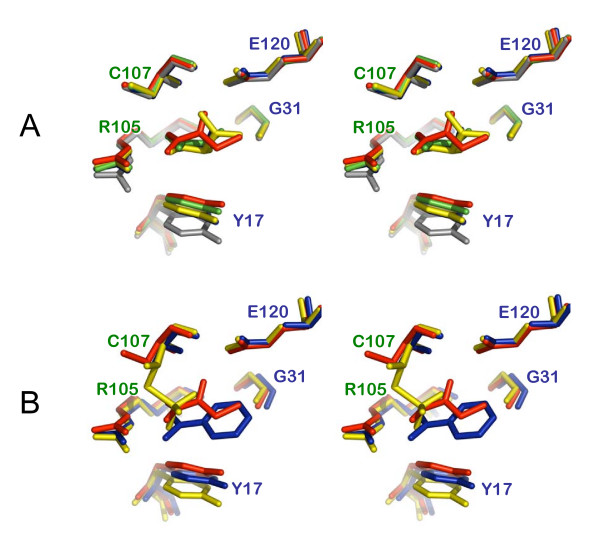
**Comparisons of the TdcF ligand-binding pocket**. Stereoviews depicting comparisons of the TdcF ligand-binding pocket. (A) shows a superposition of all the states displayed in Figures 3 and 4, where: grey = empty site; blue = ethylene glycol-bound (the ethylene glycol itself is mainly occluded by other ligands in this view); yellow = serine-bound; red = 2-ketobutyrate-bound; green = propionate-bound. (B) shows a superposition of the ligand-binding site from TdcF with 2-ketobutyrate bound in red, onto the site from hp14.5 with a benzoic acid molecule bound in blue (primary site only)(PDB accession code 1ONI), and the equivalent site in YjgF in yellow (PDB accession code 1QU9). In the latter, two conformers are seen for Cys-107, the major one being covalently modified and protruding into the ligand-binding pocket. Throughout, the view is identical to that seen in Figures 3 and 4. Figure generated using *PyMOL *[31].

**Figure 6:**
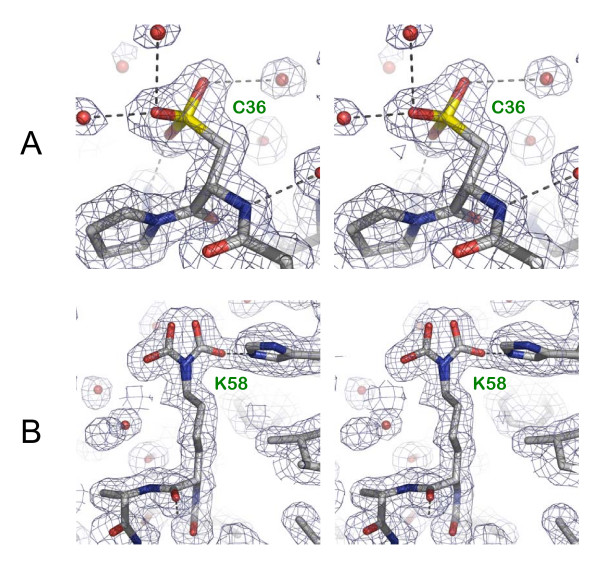
**Covalent modifications of TdcF**. Stereoviews showing covalent modifications of TdcF. In both cases, 1.6 Å resolution *2mF*_*obs *_- *dF*_*calc *_electron density maps are shown contoured at approximately 1 sigma. (A) a fully oxidised cysteine (cysteine sulfonic acid) is observed at position 36 in each subunit of all structures; (B) a carboxylated lysine in two alternative conformations that is visible in only the A chain of the 2-ketobutyrate-bound structure. Figure generated using *PyMOL *[31].

**Figure 7:**
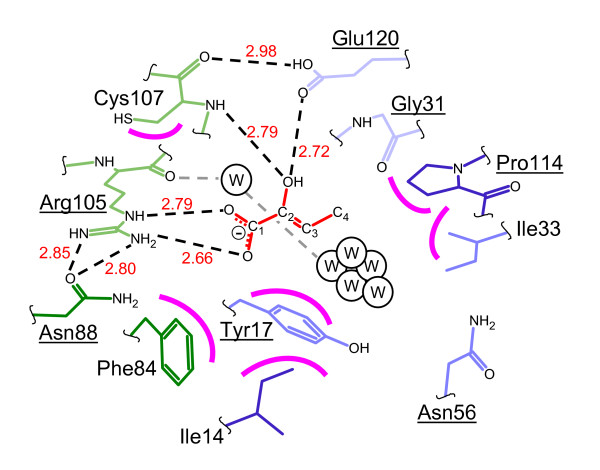
**The binding of 2-ketobutyrate to TdcF**. Schematic representation of the binding of 2-ketobutyrate to TdcF. 2-ketobutyrate is shown in red, whilst residues from one subunit are in green and those from the other subunit are in blue; darker colours indicate residues in the foreground and paler colours are used for those residues in the background. Underlining indicates strictly conserved residues in the YjgF/YER057c/UK114 family [8]. Important hydrogen bonds are shown as dashed lines and the curved magenta lines indicate non-bonding interactions with the ligand. The numbers in red give average hydrogen bond lengths in Å. Water molecules are indicated by the letter "W". The cluster of water molecules to the right of the ligand is in direct contact with bulk solvent. The view is similar to that seen in Figures 3–5.

Alignment of YjgF/YER057c/UK114 family members from different biological sources has identified seven totally conserved amino acid residues [2,6-8,20]. These conserved amino acids are found in a pocket located at the subunit interfaces of the trimer (Figure 7), and it has been suggested that they may form a substrate- or ligand-binding site. Recently, using NMR spectroscopy and a ligand-screening approach Parsons *et al*. [8] identified six compounds that interacted with the HI0719 protein from *H. influenzae *at, or near, this putative binding site. The ligand that showed the strongest interaction was 2-ketobutyrate (*K*_D_~2 mM), which is an intermediate in the biosynthetic pathway of L-isoleucine. Some 16 residues were perturbed in the ^15^N-HSQC spectrum upon binding 2-ketobutyrate, but curiously these did not include the equivalents of residues Arg-105 and Glu-120. In this study, we have demonstrated structurally that 2-ketobutyrate binds specifically to a new family member, TdcF from *E. coli*. All three ligand-binding sites were occupied in the crystal structure and the key residues identified to make hydrogen-bonding interactions with 2-ketobutyrate were Arg-105 and Glu-120, both of which are highly conserved throughout the family. Although the *2mF*_*obs *_- *dF*_*calc *_electron density maps for the side-chain of Cys-107 were always clearly defined, showing a conformation directed away from the binding pocket (see Figures 3 and 4), in most instances, there was some evidence in the difference electron density maps for one, or occasionally two, very minor alternate conformations directed either towards Arg-105 or towards the site of ligand binding. In the latter cases, this would preclude ligand binding and thus indicate less than unit occupancy for the ligand. Nevertheless, even in the vacant ligand-binding sites the predominant conformation was as shown in Figure 3A. None of these alternate conformers was modelled in the structures. Whether this residue has some role in mediating access to the pocket is not clear, but it is notable that the equivalent residue could not be clearly defined in the solution structure of HI0719 from *H. influenza*, suggesting that it might also be able to adopt different conformations [8]. The corresponding Cys-107 residue in the *E. coli *YjgF protein was observed to have a covalent modification that was proposed to be either thiosulphate or thiophosphate, perhaps also suggesting a role in ligand interaction [6]. Indeed, the modified Cys projects into the ligand-binding pocket of YjgF and hydrogen-bonds to Arg-105. In so doing, it overlaps the positions occupied by the ligands seen in the TdcF structures and thus could actually prevent ligand binding here (Figure 5B). This position is occupied by a cysteine in only about half of the TdcF orthologues currently identified. Notably, all of the orthologues predicted to bind 2-ketobutyrate or metabolites on the pathway to isoleucine biosynthesis (Hmf1 and Mmf1 in yeast [4,10,11], HI0719, in *H. influenzae *[8], YjgF in *E. coli *and *S. enterica *[2,6] have a cysteine residue at position 107.

A comparison of the four TdcF structures presented here shows that the largest changes occur between the 1.6 Å resolution structures of the as-isolated and the 2-ketobutyrate-bound forms. The rmsd value calculated for the whole trimer is 0.341 Å based on common Cα atoms. In pairwise comparisons between corresponding monomers, the largest rmsd value was for the B subunits at 0.501 Å (next largest value 0.226 Å). This was to be expected, since in the 1.6 Å resolution as-isolated structure, both the sites associated with the B monomer are empty, whilst both are full in the 2-ketobutyrate-bound structure. The largest Cα displacement of approximately 4 Å was for Ile-14 in the β1 and β2 loop (Figure 2). Recalculating the rmsd for monomer B with the exclusion of residues 11 – 17 inclusive, gave a much lower value of 0.102 Å, indicating that the conformational changes are essentially restricted to the β1 – β2 loop. In general, this loop is poorly defined and more open for the ligand-free sites, whilst in the ligand-bound sites, it closes over the ligand and becomes more ordered.

In our initial structure of TdcF, the cryoprotectant ethylene glycol was shown to occupy two of the three binding sites, but this is unlikely to be physiologically relevant. Subsequent experiments showed that propionate, L-serine and/or L-threonine could also occupy the binding sites. However, again not all of the binding sites were occupied. By contrast, full site occupancy was achieved using 2-ketobutyrate, indicating that it binds with higher affinity than the other ligands. Taken together, these results strongly suggested that the subunit interface represents a binding site for a ligand or substrate that is an intermediate in the metabolism of L-threonine or L-serine. This is also in agreement with findings for other orthologues where it has been proposed that 2-ketobutyrate, or a metabolic derivative thereof, interacts with the binding site [2,3,8].

We and others [6-8,10,20] have analysed the ligand-binding pocket in detail, and through comparisons with other known structures have attempted to predict a catalytic function. Although the fold of TdcF resembles that of chorismate mutase and the 2-ketobutyrate-binding pocket maps directly onto the active site of this enzyme, the important functional groups within the pocket are not conserved with this enzyme. In addition, some sequence similarity to 2-aminomuconate deaminases has been noted [8], significantly, the equivalents of Arg-105 and Glu-120 being conserved, but when HI0719 was tested for 2-aminomuconate deaminase activity, it proved to be inactive. The observation of a water molecule stacked against one face of the enol moiety of the 2-ketobutyrate is intriguing as it could take part in catalysis. In addition, the cluster of water molecules adjacent to the 2-ketobutyrate (Figure 4A and Figure 7) could indicate a binding site for another substrate, or perhaps together with the space occupied by the 2-ketobutyrate, may represent the binding site for a much larger ligand. Another possibility is that a cofactor binds adjacent to the 2-ketobutyrate. Crude docking experiments with our TdcF structures suggest that a pyridoxal phosphate molecule could be accommodated, whilst thiamine pyrophosphate or coenzyme A (CoA) could not. Nevertheless, we cannot rule out the possibility that the latter two ligands induce large conformational changes that allow them to bind. However, investigations with HI0719 showed that none of these cofactors induced chemical shifts in the ^15^N-HSQC spectrum of this protein [8].

## Conclusion

The findings presented in this study strongly support the contention that 2-ketobutyrate is a physiological ligand recognised by TdcF. The *tdcF *gene is located in a multi-cistronic operon whose gene products have a role in the anaerobic degradation of L-threonine and L-serine. The first intermediate in the degradation of L-threonine or L-serine is the ketoacid, 2-ketobutyrate or pyruvate, respectively. Pyruvate can be further metabolised via pyruvate formate-lyase or pyruvate dehydrogenase to acetyl-CoA. 2-ketobutyrate can either be used as a substrate on the pathway to L-isoleucine or, under anaerobic conditions, can be metabolised to propionate via propionyl-CoA and propionyl-P intermediates with concomitant generation of 1 ATP (Figure 1). All the prerequisite enzymes for this fermentative route, with the exception of phosphotransacetylase are encoded by the *tdc *operon [15]. The only gene product of the operon whose function could not yet be assigned is TdcF. Since to date no enzyme activity has been detected for any member of this protein family, we suggest, based on the current findings, that TdcF may be a post-translational regulator that controls the metabolic fate of L-threonine or the potentially toxic intermediate 2-ketobutyrate. Depending on whether L-isoleucine is limiting or not for growth, TdcF, by sensing the levels of 2-ketobutyrate and forming a ternary complex with one or more of the enzymes of isoleucine biosynthesis or 2-ketobutyrate degradation, ensures that 2-ketobutyrate does not accumulate in the cell. Experiments to determine putative protein-protein interaction partners of TdcF are in hand. A similar proposal for the function of the members of this protein family has been made previously [2,3]. This is consistent with the recent demonstration that YjgF appears to function at the post-translational level in controlling the activity of IlvE, which catalyses the final transamination step on the L-isoleucine pathway in *Salmonella enterica *[3]. The accumulation of 2-ketobutyrate has been proposed [23], and genetically demonstrated [13], to compete with 2-ketoisovalerate, the precursor of pantothenate synthesis, resulting in starvation for coenzyme A. Metabolic poisoning by 2-ketobutyrate is prevented by its degradation aerobically via pyruvate dehydrogenase [14] and anaerobically via pyruvate formate-lyase or TdcE [15]; these reactions are CoA-dependent. This role of a sensor of 2-ketobutyrate would not only afford protection to cells from the toxic effects of 2-ketobutyrate accumulation and provide an additional means of energy generation, but also would ensure that sufficient 2-ketobutyrate was available for L-isoleucine biosynthesis.

## Methods

### Purification and crystallisation

Overproduction, purification and crystallisation of the recombinant, N-terminally His-tagged, TdcF protein was performed exactly as described previously [17]. The His-tag was not cleaved prior to crystallisation. Briefly, crystals were obtained using the hanging drop vapour diffusion method with a 1:1 mixture of protein (10 mg/ml in 20 mM Tris-HCl, pH 8) and well solution (12.5% w/v PEG 1500). Needle-like crystals appeared after a week, although in some drops, more substantial, rectangular, crystals formed over a period of up to 2 months, having a maximum size of approximately 150 × 60 × 60 μm. Crystals were routinely transferred from one solution to another, and ultimately mounted for X-ray data collection, using cryo-loops (Hampton research). For the first data set, crystals were cryoprotected by soaking for a maximum of 5 min in crystallisation solution supplemented with 20% (w/v) ethylene glycol. However, after discovering ethylene glycol bound to the protein, 20% (w/v) PEG 400 was substituted as the cryoprotectant.

### Crystal soaking

For ligand soaking experiments, crystals were transferred from the mother liquor to a soaking solution comprising 12.5% (w/v) PEG 1500 in 20 mM Tris-HCl pH8 and containing 1 mM of the potential ligand molecule. The latter were chosen on the basis of being substrates, products or metabolic intermediates on the L-serine/L-threonine degradation pathways. These included L-threonine, L-serine, pyruvate, propionate, 2-ketobutyrate and propionyl-CoA. Crystals were soaked for 2 h before being cryoprotected in fresh soaking solution containing the ligand and 20% (v/v) PEG 400.

### X-ray data collection and processing

For in-house data collection, crystals were flash-cooled and maintained at 100 K using an X-Stream cryocooler (Rigaku-MSC) and X-ray data were recorded on a Mar 345 image plate detector (X-ray Research) mounted on a Rigaku RU-H3RHB rotating anode X-ray generator (operated at 50 kV and 100 mA) fitted with Osmic confocal optics and a copper target (Cu *Kα *; λ = 1.542 Å).

For synchrotron data collection, crystals were flash-cooled by plunging into liquid nitrogen and stored prior to transport to the synchrotron. Crystals were transferred to the goniostat on station ID14-2 (λ = 0.933 Å) at the European Synchrotron Radiation Source (ESRF) in Grenoble using Hampton Research cryotools and maintained at 100 K with a Cryostream cryocooler (Oxford Instruments). Diffraction data were recorded on an ADSC Quantum 4 CCD.

All X-ray data were processed using version 1.97 of the *HKL *software package [24], and downstream processing and statistical analysis was effected using programs from the CCP4 suite [25]. Data collection statistics are summarised in Table 2.

### Structure solution and refinement

The space group of the TdcF crystals was *P*2_1_2_1_2, with approximate cell parameters of a = 72, b = 86 and c = 63 Å. Solvent-content estimation based on a TdcF trimer in the asymmetric unit gave a *V*_*M *_value of 2.02 Å^3 ^Da^-1^, corresponding to a solvent content of 39%.

Molecular replacement was performed using the program *AMoRe *[26] with the structure of the closely-related YjgF [6] as a template; this was successful in placing a trimer in the asymmetric unit [17]. Model building was performed by interactive computer graphics using the program *O *[27] by inspection of *2mF*_*obs*_*- dF*_*calc *_and *mF*_*obs*_*- dF*_*calc *_Fourier electron density maps. Ligands were docked with reference to unbiased *mF*_*obs*_*- dF*_*calc *_difference maps. All data sets were essentially isomorphous and an equivalent subset of the data comprising 5% of the reflections was set aside for the calculation of 'free' (R_free_) crystallographic R-factors [28] during model refinement. Throughout refinement, neither low resolution nor amplitude cut-offs were applied. Both positional and thermal parameters of the models were subsequently refined using *REFMAC5 *[29]. Anisotropic thermal parameters were refined for the two structures at 1.6 Å resolution. A summary of the model contents and geometrical parameters of the final structures are given in Table 2. The coordinates and structure factor data for these structures have been deposited in the Protein Data Bank.

## Authors' contributions

RGS and DML conceived the study and together with JDB, drafted the manuscript. All experimental work was performed by JDB, with assistance from RGS for the expression and purification; and with assistance from CEMS and DML for the X-ray crystallographic analysis. All authors have approved the final manuscript.

## Figures and Tables

**Table 1:** Summary of TdcF structural homologues

**Protein**	**Source**	**Identity (%)**^a^	**Aligned residues**	**PDB code**	**Resolution (Å)**	**Rmsd (Å)**^b^	**Reference**
TdcF	*E. coli*	100	-	2UYK	1.60	0.0	this work
YjgF	*E. coli*	73	126	1QU9	1.20	0.5	[6]
HI0719	*H. influenzae*	63	126	1J7H	NMR	1.4	[8]
PTII^c^	*C. thermocellum*	51	122	1XRG	2.20	1.1	to be published
TM0215	*T. maritima*	49	124	2B33	2.30	1.0	to be published
YabJ	*B. subtilis*	49	123	1QD9	1.70	1.1	[7]
APE1501	*A. pernix*	46	115	2CWJ	3.60	1.3	to be published
UK114	*C. hircus*	44	124	1NQ3	2.20	1.2	[19]
hp14.5^d^	*H. sapiens*	43	124	1ONI	1.90	1.1	[9]
L-PSP	*R. norvegicus*	43	124	1QAH	1.80	1.0	to be published
ST0811	*S. tokodaii*	41	123	1X25	2.00	0.9	[20]
TTHA0137^e^	*T. thermophilus*	40	121	2CVL	1.65	1.2	to be published
Hmf1	*S. cerevisiae*	35	123	1JD1	1.70	1.1	[10]
YjgH	*E. coli*	31	122	1PF5	2.50	1.8	to be published
PA3499	*P. aeruginosa*	27	120	2IG8	1.90	1.6	to be published

^a ^Identity versus TdcF in a structure-based sequence alignment.

^b ^Comparisons made between the TdcF as-isolated 1.6 Å resolution structure and its homologues. Only the Cα atoms of monomers (A chains) are compared, and in the case of HI0719 (the only NMR structure), the comparison is with chain A of model 1 from a 20 model ensemble.

^c ^PTII = putative translation initiation inhibitor

^d ^Besides TdcF, this is the only other structure of a homologue to contain a biologically relevant ligand, namely benzoic acid.

^e ^There are in fact three PDB entries for this protein (also 2CSL and 2CW4), which differ in space group. Only the highest resolution structure is shown.

**Table 2:** Summary of X-ray data and model parameters for TdcF

**Data set**	**As-isolated (ethylene glycol)**	**As-isolated (PEG 400)**	**2-Ketobutyrate soak**	**Propionate soak**
*Data collection*				
Radiation source	RU-H3RHB	1D14-2 ESRF	1D14-2 ESRF	RU-H3RHB
Wavelength (Å)	1.542	0.933	0.933	1.542
Cell parameters (Å)	a = 72.7 b = 86.2 c = 62.6	a = 72.7 b = 86.4 c = 62.6	a = 72.6 b = 85.9 c = 62.7	a = 72.4 b = 85.7 c = 62.5
Resolution range^a ^(Å)	23.7 – 2.35 (2.43 – 2.35)	35.9 – 1.60 (1.63 – 1.60)	35.4 -1.60 (1.63 – 1.60)	25.5 – 2.45 (2.54 – 2.45)
Unique reflections	16863	50526	49876	14841
Completeness^a ^(%)	99.3 (95.2)	95.2 (66.4)	94.9 (71.2)	98.9 (94.0)
Redundancy	4.4	2.9	3.3	3.4
R_merge _^a,b^	0.103 (0.368)	0.071 (0.116)	0.060 (0.243)	0.077 (0.303)
<I/σ (I)>^a^	12.1 (2.7)	19.1 (3.8)	20.5 (2.1)	14.6 (3.2)
Wilson B value (Å^2^)	29.5	15.8	17.1	35.9
*Refinement*				
R_cryst _^c ^(based on 95% of data; %)	17.2	15.5	16.2	16.6
R_free _^c ^(based on 5% of data; %)	24.4	19.9	20.4	25.3
DPI^d ^(based on R_free_; Å)	0.257	0.087	0.089	0.295
Ramachandran plot^e ^(%)	94.0	95.8	95.5	94.6
Rmsd bond distances (Å)	0.020	0.017	0.015	0.021
Rmsd bond angles (°)	1.800	1.555	1.441	1.843
*Contents of model*				
Protein (residues/atoms)	383/2876	381/2886	381/2895	382/2860
Waters (molecules)	205	457	400	195
Ligands (molecules/atoms)	2/8	1/7	3/21	2/10
Ligands (identity^f^/sites occupied)	EDO/B,C	SER/C	2KT/A,B,C	PPI/B,C
*Average temperature factors (Å^2^)*				
Main-chain atoms	17.7	13.0	14.9	21.0
Side-chain atoms	18.5	15.6	17.4	21.6
Waters	23.1	29.8	30.8	26.8
Ligands	29.4	19.2	24.7	33.8
Overall	18.4	16.4	17.9	27.7
PDB accession code	2UYJ	2UYK	2UYN	2UYP

^a ^The figures in brackets indicate the values for outer resolution shell.

^b ^R_merge _= Σ_h_Σ_*l*_|*I*_h*l *_- <*I*_h_>|/Σ_h_Σ_*l *_<*I*_h_>, where *I*_*l *_is the *l*^th ^observation of reflection h and <*I*_h_> is the weighted average intensity for all observations *l *of reflection h.

^c ^The R-factors R_cryst _and R_free _are calculated as follows: R = Σ (|F_obs _- F_calc_|)/Σ |F_obs_| × 100, where F_obs _and F_calc _are the observed and calculated structure factor amplitudes, respectively.

^d ^Diffraction-component precision index [32] – an estimate of the overall coordinate errors calculated in *REFMAC5 *[29].

^e ^Residues with most favoured Φ/Ψ angles as calculated using *PROCHECK *[33].

^f ^EDO = ethylene glycol (1,2-ethanediol); 2KT = 2-ketobutyrate; PPI = propionate.
